# A comprehensive assessment of single nucleotide polymorphisms associated with pancreatic cancer risk: A protocol for systematic review and network meta-analysis

**DOI:** 10.1097/MD.0000000000020345

**Published:** 2020-06-12

**Authors:** Zhuo-Miao Ye, Li-Juan Li, Jing-Hui Zheng, Chi Zhang, Yun-Xin Lu, Youming Tang

**Affiliations:** a Ruikang School of Clinical Medicine, Guangxi University of Chinese Medicine; b The First Clinical Faculty of Guangxi University of Chinese Medicine; c Department of Cardiology, Ruikang Hospital Affiliated to Guangxi University of Chinese Medicine, Nanning; d Graduate School, Guangxi University of Chinese Medicine; e Department of Oncology; f Department of Gastroenterology, Ruikang Hospital Affiliated to Guangxi University of Chinese Medicine, Nanning, China.

**Keywords:** case-control study, model of inheritance, network meta-analysis, pancreatic cancer, susceptibility

## Abstract

**Background:**

Single nucleotide polymorphisms (SNPs) have been inconsistently associated with pancreatic cancer (PC) risk. This meta-analysis aimed to synthesize relevant data on SNPs associated with PC.

**Methods:**

Databases were searched to identify association studies of SNPs and PC published through January 2020 from the databases of PubMed, Web of Science, Embase, Cochrane Library, China National Knowledge Infrastructure, the Chinese Science and Technology Periodical Database (VIP) and Wanfang databases. Network meta-analysis and Thakkinstian algorithm were used to select the most appropriate genetic model, along with false positive report probability (FPRP) for noteworthy associations. The methodological quality of data was assessed based on the STREGA statement Stata 14.0 will be used for systematic review and meta-analysis.

**Results:**

This study will provide a high-quality evidence to find the SNP most associated with pancreatic cancer susceptibility and the best genetic model.

**Conclusions:**

This study will explore which SNP is most associated with pancreatic cancer susceptibility.

Registration: INPLASY202040023.

## Abstract 2

Supplemental Digital Content is available in the text

## 1 Introduction

Pancreatic Cancer (PC) is the deadliest malignant tumor and the eighth leading cause of cancer-related death in the world, with a 1-year survival rate of less than 5%.^[1]^ Epidemiological risk factors for pancreatic cancer, including smoking, heavy drinking, diabetes, obesity, chronic pancreatitis, and a family history of pancreatic cancer, have been identified in the current world. Genetic factors play an important role in the etiology of pancreatic cancer.^[2]^ Studies have found that there are many genes associated with pancreatic cancer susceptibility, such as TERT, UGT2B4, XRCC4, XPC, SLC22A3, NR5A2, ABO, and XPD gene mutation makes people susceptible to pancreatic cancer.^[3–10]^ Thus, pancreatic cancer is a kind of gene-environment interaction of genetic mutations in complex diseases, including single nucleotide polymorphism is an important part of individual genetic variation, the fact that encourages single nucleotide polymorphisms (SNPs) and the risk of pancreatic cancer in such correlation research, To determine which genes are more susceptible to pancreatic cancer.^[11]^ SNPs represent the most common type of variation in the human genome. The SNPs located in protein-coding and non-coding RNA genes are classified as neutral and functional.^[12]^ NPS have been found to alter gene expression and function, or to produce linkage imbalances at causal sites associated with cancer risk and/or prognosis. Such as insulin-like growth factor, genetic variants in the platelet-derived growth factor subunit B gene, variants in atopy-related immunologic candidate genes, taste-related genes, inflammatory genes, it is thought to affect an individual's susceptibility to pancreatic cancer.^[5,13–17]^ Most of these studies, however, have limited statistical power to detect small-effect SNPs and the results are often inconsistent and thus inconclusive. Building upon these studies, systematic reviews have evaluated the evidence regarding SNPs in individual genes or signaling pathways related to pancreatic cancer.^[18–21]^ But few reviews have comprehensively summarized and evaluated all SNPs related to pancreatic cancer. The aim of this study was to assess the significance of SNPs in pancreatic cancer susceptibility in populations worldwide. At the same time, without assuming the underlying genetic model, we used various methods to select the most appropriate genetic model and to measure the reliability of the association to find out which gene model was most suitable for identifying the association between SNPs and pancreatic cancer.

## 2 Objective

The objective of this study was to comprehensively evaluate significant SNPs associated with PC susceptibility. Moreover, we aim to indicate which genetic model is most appropriate to identify associations of SNPs with PC.

## 3 Methods

The methods of this systematic review conducted in accordance with the preferred reporting items for systematic reviews and meta-analyses (PRISMA) guidelines and the protocol has been registered in the INPLASY database.

### 3.1 Criteria for the included studies in the review

#### 3.1.1 Types of studies

Case-control study related to the susceptibility of the SNPs to the PC will be incorporated in our review. Repeat report, conference report, thesis, review paper, or animal study, or study has insufficient data for genotyping distribution calculation or which SNPs demonstrated a departure from Hardy-Weinberg equilibrium in controls were excluded.

#### 3.1.2 Participants

Participants affected by PC and were taken serum samples before prior chemoradiotherapy will be included in the meta-analysis. Noncancer controls may be healthy or have non-malignant diseases. No restrictions were placed on age, gender, country, or tumor stage.

#### 3.1.3 Outcome

Pancreatic risk comparsions.

### 3.2 Search strategy

#### 3.2.1 Electronic searches

We will search for relevant studies in the following databases: PubMed, Web of Science, Embase, Cochrane Library, China National Knowledge Infrastructure, the Chinese Science and Technology Periodical Database (VIP) and Wanfang databases, with no language limits. All those studies published through January 2020. The search strategy was based on the following search terms: “single nucleotide polymorphism,” “SNP,” “pancreatic cancer,” and “Pancreatic Neoplasm.” Details regarding the search terms are available in the Supplementary Material 1.

### 3.3 Data collection

#### 3.3.1 Selection of studies

Apart of the authors in our team will be trained regarding the purpose and process of the review. The selection work will require 3 independent authors. Two reviewers (ZY and LL) conducted the selection process independently, with cases of disagreement resolved by discussion or consulting a third reviewer (JZ).

**Figure 1:**
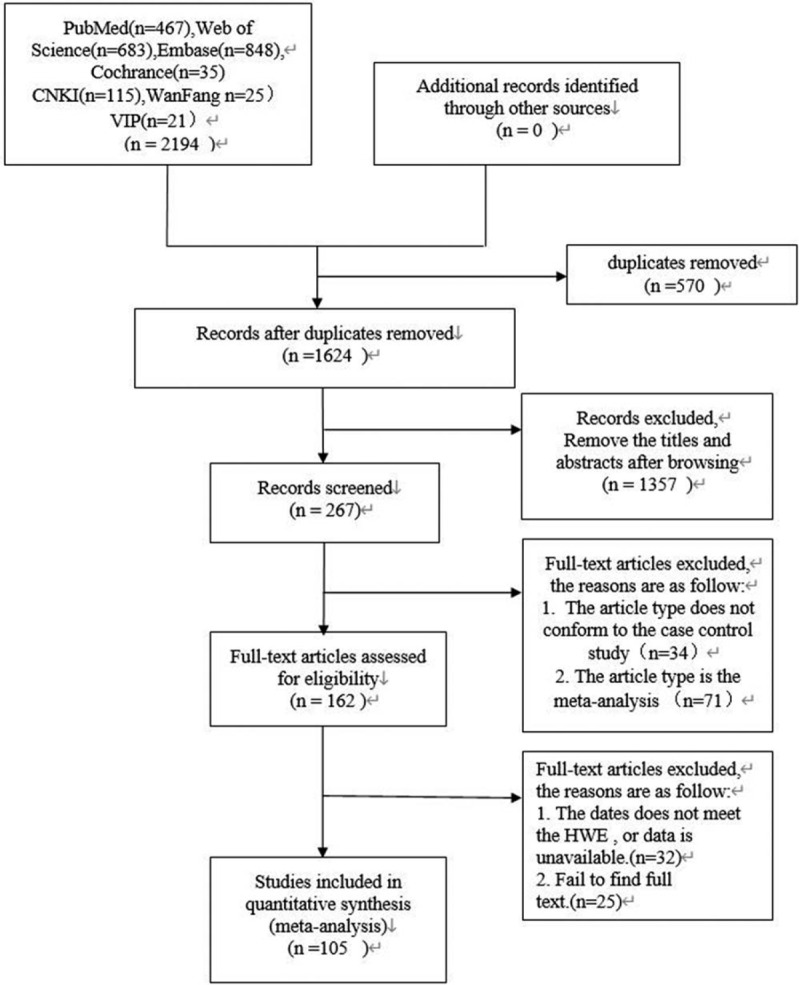
PRISMA flow diagram of literature search and selection.

Figure 1 is the PRISMA flow diagram illustrating the procedure of study selection.

#### 3.3.2 Data extraction and qualitative evaluation

Data extracted from individual papers include: author, year of publication, country, sample size, the value of Hardy-Weinberg equilibrium, sex composition, age of diagnosis, and details of target SNPs, including genotyping methods, frequencies of genotypes. The methodological quality of data was assessed based on the STREGA statement.^[22]^ Two reviewers (ZY and LL) conducted the rating independently and a third reviewer (JZ) was consulted for consensus if disagreement occurred.

#### 3.3.3 Dealing with missing data

We will attempt to contact the corresponding authors if the data of potential studies are missing, insufficient, or vague. However, the studies will be excluded if we cannot obtain the relevant data via the aforementioned approaches.

#### 3.3.4 Statistical analysis

StataMP14.0 software will be used to analyze these data. We calculated fixed- or random-effects pooled odds ratio (OR) with 95% confidence intervals (CIs) for pairwise meta-analysis, depending on degree of heterogeneity under different genetic models (allele contrast model, homozygous model, heterozygous model, dominant model, recessive model).

#### 3.3.5 Assessment of heterogeneity

Heterogeneity was quantified with the I^2^ statistic and *P* value; a I^2^ statistic < 50% and a *P* > .1 indicated low heterogeneity between studies, in which case the fixed-effect model was employed, otherwise, random effects model will be used. For significant SNPs with evidence of heterogeneity in meta-analysis, assessment of sources of heterogeneity was employed using subgroup analysis if sufficient data existed.

#### 3.3.6 Assessment of reporting biases

We will analyze the potential publication bias by generating funnel plots if the number of the study is enough (> = 10). Publication bias was assessed using the Begg and Egger tests.

#### 3.3.7 Network meta-analysis

A random-effects network meta-analysis within a Bayesian framework was conducted using the GeMTC software (v 0.14.3).^[23]^ Four parallel Markov chain Monte Carlo simulations were run for a 20,000-stimulation burn-in phase and an additional 50,000-stimulation phase. Convergence was satisfied with a potential scale reduction factor value of 1.0 as the cut-off value. Consistency, referring to agreement between direct and indirect comparisons in terms of effect estimates, was evaluated by comparing consistency model with inconsistency model in terms of standard deviation of the random effect. The inconsistency model was used when an obvious deviation was detected; otherwise, the consistency model was used. This Bayesian approach was used to rank the probability of each genetic model for risk assessment for PC and corresponding rank probability plots were generated.

#### 3.3.8 False positive report probability (FPRP)

We further compared genetic models to select the most appropriate model using the algorithm by Thakkinstian et al.^[24]^ To assess the noteworthiness of the normally significant SNPs under the most appropriate genetic model determined by network meta-analysis or Thakkinstian’ algorithm, false positive report probability (FPRP) was calculated assuming three levels of prior probabilities (low: 0.1; moderate: 0.01; high: 0.001) and an OR of 1.5, as previously described.^[25,26]^ Significant SNPs with a FPRP value < 0.2 were considered noteworthy.^[25]^

#### 3.3.9 Diagnostic meta-analysis

Diagnostic meta-analysis was conducted to determine sensitivity and specificity of SNPs in predicting PC risk using the Meta-DiSc software^[27]^ just as Zhang's study did.^[28]^

#### 3.3.10 Subgroup analysis

We will conduct a subgroup analysis of the SNPs most associated with pancreatic cancer, according to race, type of virus infection, age, sex, etc.

#### 3.3.11 Sensitivity analysis

Sensitivity analysis will be conducted to check the robustness and reliability of pooled outcome results.

#### 3.3.12 Assessment of publication biases

We will evaluate publication bias using the funnel plot as well as statistical tests (Egger test and Begg test).

### 3.4 Discussion

Risk association analysis based on a priori genetic model may be misleading if an inappropriate genetic model was assumed.^[28]^ Several decades of intense research have generated large amounts of data on the genetic susceptibility of PC, yet the empirical findings have been mixed and inconclusive regarding PC susceptibility related to SNPs. In this study, we conducted a meta-analysis to combine findings from multiple studies and generate a more robust estimate of risk association to assess the current state of research on this topic. This is the first systematic review and meta-analysis to our knowledge to comprehensively assesse SNPs associated with PC In the study of correlation in PC risk, SNPs are effective methods to evaluate gene-gene and gene-environment interactions. Risk association analysis based on a priori genetic model can be misleading if an inappropriate genetic model is postulated. Therefore, by the end of our literature search in February 2020, we collected 310 SNPs. This study did not make any assumptions, and observed the genotype significance of which gene models for PC susceptibility in a paired meta-analysis. To determine the most appropriate PC risk association model, network meta-analysis and Thakkinstian algorithms were used. Those SNPs we obtained through analysis of our study may assist clinicians in assessing the prognosis of PC patients and selecting appropriate targets therapy.^[29]^ Our meta-analysis of genes for PC susceptibility factors requires additional large sample size, detailed PC risk factor data and high-quality studies to further assess the role of gene-gene and gene-environment interactions in determining PC risk.

## Author contributions

**Analysis planning:** Jing-Hui Zheng, Yun-Xin Lu

**Conceptualization:** Jing-Hui Zheng, Yun-Xin Lu

**Data curation:** Zhuo-Miao Ye, Li-Juan Li

**Draft manuscript:** Zhuo-Miao Ye, Li-Juan Li

**Investigation:** Jing-Hui Zheng, You-Ming Tang

**Manuscript editing:** Zhuo-Miao Ye, Jing-Hui Zheng

**Methodology:** Zhuo-Miao Ye, Jing-Hui Zheng, Yun-Xin Lu

## Supplementary Material

Supplemental Digital Content
