# Supplementary material for: A comprehensive assessment of single nucleotide polymorphisms associated with pancreatic cancer risk: A protocol for systematic review and network meta-analysis
Source: Medicine (Baltimore). 2020 Jun 12;99(24):e20345. doi: 10.1097/MD.0000000000020345 (PMC7302655; doi:10.1097/MD.0000000000020345)
Supplement: Supplemental Digital Content [file medi-99-e20345-s001.docx]

**Pubmed：**

Search ((((((((Pancreatic Neoplasm[Title/Abstract]) AND (((((((Single Nucleotide Polymorphisms[Title/Abstract]) OR Nucleotide Polymorphism, Single[Title/Abstract]) OR Nucleotide Polymorphisms, Single[Title/Abstract]) OR Polymorphisms, Single Nucleotide[Title/Abstract]) OR Single Nucleotide Polymorphisms[Title/Abstract]) OR SNPs[Title/Abstract]) OR Single Nucleotide Polymorphism[Title/Abstract]))) OR ((Neoplasms, Pancreas[Title/Abstract]) AND (((((((Single Nucleotide Polymorphisms[Title/Abstract]) OR Nucleotide Polymorphism, Single[Title/Abstract]) OR Nucleotide Polymorphisms, Single[Title/Abstract]) OR Polymorphisms, Single Nucleotide[Title/Abstract]) OR Single Nucleotide Polymorphisms[Title/Abstract]) OR SNPs[Title/Abstract]) OR Single Nucleotide Polymorphism[Title/Abstract]))) OR ((Pancreas Cancer[Title/Abstract]) AND (((((((Single Nucleotide Polymorphisms[Title/Abstract]) OR Nucleotide Polymorphism, Single[Title/Abstract]) OR Nucleotide Polymorphisms, Single[Title/Abstract]) OR Polymorphisms, Single Nucleotide[Title/Abstract]) OR Single Nucleotide Polymorphisms[Title/Abstract]) OR SNPs[Title/Abstract]) OR Single Nucleotide Polymorphism[Title/Abstract]))) OR ((Cancers, Pancreas[Title/Abstract]) AND (((((((Single Nucleotide Polymorphisms[Title/Abstract]) OR Nucleotide Polymorphism, Single[Title/Abstract]) OR Nucleotide Polymorphisms, Single[Title/Abstract]) OR Polymorphisms, Single Nucleotide[Title/Abstract]) OR Single Nucleotide Polymorphisms[Title/Abstract]) OR SNPs[Title/Abstract]) OR Single Nucleotide Polymorphism[Title/Abstract]))) OR ((Pancreatic Cancer[Title/Abstract]) AND (((((((Single Nucleotide Polymorphisms[Title/Abstract]) OR Nucleotide Polymorphism, Single[Title/Abstract]) OR Nucleotide Polymorphisms, Single[Title/Abstract]) OR Polymorphisms, Single Nucleotide[Title/Abstract]) OR Single Nucleotide Polymorphisms[Title/Abstract]) OR SNPs[Title/Abstract]) OR Single Nucleotide Polymorphism[Title/Abstract]))) OR ((Cancers, Pancreatic[Title/Abstract]) AND (((((((Single Nucleotide Polymorphisms[Title/Abstract]) OR Nucleotide Polymorphism, Single[Title/Abstract]) OR Nucleotide Polymorphisms, Single[Title/Abstract]) OR Polymorphisms, Single Nucleotide[Title/Abstract]) OR Single Nucleotide Polymorphisms[Title/Abstract]) OR SNPs[Title/Abstract]) OR Single Nucleotide Polymorphism[Title/Abstract]))) OR ((Pancreatic Cancers[Title/Abstract]) AND (((((((Single Nucleotide Polymorphisms[Title/Abstract]) OR Nucleotide Polymorphism, Single[Title/Abstract]) OR Nucleotide Polymorphisms, Single[Title/Abstract]) OR Polymorphisms, Single Nucleotide[Title/Abstract]) OR Single Nucleotide Polymorphisms[Title/Abstract]) OR SNPs[Title/Abstract]) OR Single Nucleotide Polymorphism[Title/Abstract]))
